# From policy to practice: prioritizing person-centred healthcare actions in the state of Victoria

**DOI:** 10.1186/s12961-021-00782-2

**Published:** 2021-10-26

**Authors:** Peter Bragge, Lidia Horvat, Louise Mckinlay, Kim Borg, Belinda Macleod-Smith, Breanna Wright

**Affiliations:** 1 BehaviourWorks Australia, Monash Sustainable Development Institute, Monash University, 8 Scenic Boulevard, Clayton Campus, Victoria, 3800 Australia; 2 Department of Health and Human Services, Safer Care Victoria, 50 Lonsdale Street, Melbourne, VIC 3000 Australia

**Keywords:** Patient-centred care, Health policy, Prioritization, Codesign

## Abstract

**Background:**

Meaningful involvement of consumers in healthcare is a high priority worldwide. In Victoria, Australia, a Partnering in Healthcare (PiH) policy framework was developed to guide health services in addressing consumer-focused healthcare improvements. The aim of this project was to identify priorities for improvement relating to the framework from the perspective of Victorian healthcare consumers and those who work in the healthcare sector.

**Methods:**

A survey of Victorians representing key stakeholder groups was used to identify a “long list” of potential priorities, followed by a day-long summit to reduce this to a “short list” using explicit prioritization criteria. The survey was piloted prior to implementation, and diverse consumer groups and key health service providers were purposefully sampled for the summit.

**Results:**

The survey (*n* = 680 respondents) generated 14–20 thematic categories across the proposed framework’s five domains. The summit (*n* = 31 participants, including *n* = 21 consumer representatives) prioritized the following five areas based on the survey findings: communication, shared decision-making, (shared) care planning, health (system) literacy and people (not) around the patient. These priorities were underpinned by three cross-cutting principles: care/compassion/respect, accountability and diversity.

**Conclusion:**

Few studies have explicitly sought consumer input on health policy implementation. Adopting a codesign approach enabled the framework to be a shared foundation of healthcare improvement. The framework was subsequently launched in 2019. All Victorian health services are required to commit annually to improvement priorities against at least two framework domains.

**Supplementary Information:**

The online version contains supplementary material available at 10.1186/s12961-021-00782-2.

## Background

Efforts to meaningfully engage patients, clinicians and other healthcare end-users in health research, design, delivery and evaluation has been a high priority around the world this century [1–4]. Victoria, Australia, has a long-standing history of policy frameworks that have supported greater consumer participation in healthcare. Hospital-level healthcare in Victoria is provided by 57 metropolitan and 69 rural hospitals and health services [5].

Consistent with worldwide trends, a number of inquiries conducted in Victoria in recent years have highlighted the need to improve patient participation in healthcare. These included a review of consumer participation in the health system conducted by the Victorian Auditor-General’s Office in 2012 [6], the Doing it with us not for us: Strategic Direction 2010–2013 [7] and the 2009 Cultural Responsiveness Framework [8]. More recently, a 2016 review of hospital safety and quality assurance in Victoria recommended patient engagement and patient experience as a priority improvement goal for the hospital system [9]. This focus is also recognized from a compliance perspective, as evidenced by the national accreditation standards for public hospitals, where “Partnering with Consumers” underpins all other standards [10].

In January 2017, Safer Care Victoria (SCV) was established as the peak Victorian government authority for quality and safety improvements in healthcare. As well as monitoring the standards of care provided, a major remit of SCV is partnering with consumers and their families, clinicians and health services to support continuous healthcare improvement [11]. The Partnering in Healthcare (PiH) policy framework is a major initiative of SCV. *Partnering in healthcare—for better care and outcomes* outlines a codesign approach to develop a healthcare framework for consumer participation. The aims of this framework are to strengthen person- and family-centred healthcare and improve healthcare experiences and outcomes for all Victorians.

Designed to help healthcare services respond to consumer needs and expectations, the PiH framework was initially designed after reviewing best available research, evidence and practice. This resulted in five interdependent domains, which formed the basis of the current research. The five domains were initially conceptualized as:

1. Person- and family-centred services, care and outcomes
2. Teams, partnerships, knowledge and shared learnings
3. Participation and shared decision-making
4. Equity, diversity, inclusion and responsiveness
5. Health literacy, information and communication.

Each domain is designed to enable action in a practical way at a direct care level (e.g. through structured shared decision-making aids and practices); a service level (e.g. through involvement in the design and delivery of healthcare services); and a system level (e.g. policy development, governance and performance monitoring) [12, 13].

As part of their policy implementation plan, SCV engaged BehaviourWorks Australia (BWA) to identify stakeholders’ priorities to address the five PiH domains. Specifically, the aim of this project was to use a codesign approach to identify priorities for improvement relating to the initial five domains in the proposed framework. A broad range of stakeholder groups were invited to contribute their perspectives on the proposed framework and priorities relevant to PiH, including policy-makers, health professionals and researchers. Given the intent of the framework, particular emphasis was placed on views of healthcare consumers, defined as follows:

Patients, consumers, families, carers, clients, residents and communities who are current or potential users of healthcare services. This includes children, women and men, people living with a disability, people from diverse cultural and religious experiences, socioeconomic status and social circumstances, sexual orientations, sex, gender and gender identity, health and illness conditions. [7]

This paper presents the findings of two prioritization activities to achieve the above aim—a large statewide survey followed by a day-long prioritization summit.

## Methods

A review of codesign literature previously published by a member of the research team (PB) illustrated that codesign approaches exist along a spectrum from representation of consumers in some activities to deeper coproduction partnerships which involve consumers’ input into research design, conduct and publication [14]. In relation to this spectrum, the present project actively involved consumers in multiple activities, including having a consumer (an employee of SCV) on the authorship team (BMS). In particular, the focus of the workshop was participatory rather than didactic/passive.

The research team worked in close collaboration with SCV, drawing upon established prioritization principles and processes applied to healthcare across a range of previously published projects [15–19]. Key principles underlying this approach are:

- Initial scoping with the organization/client to determine what is being prioritized and the target number of priorities that are realistic to address
- Identify all groups that have a stake in the area and engage widely with representatives of these groups to create an exhaustive “long list” of potential priorities
- Prioritize a “short list” (target number of priorities) from the long list through further engagement with the same representative group
- Use explicit prioritization criteria and make the target for the short list and the rationale for this clear to participants in the process
- Communicate findings of prioritization widely to all groups and individuals that have participated in the prioritization process.

Underpinned by these principles and organized around the five domains of the proposed PiH framework, this project comprised:

1. a statewide survey of Victorians to identify a long list of potential priorities
2. a day-long prioritization summit to reduce this to a short list using explicit prioritization criteria.

The research was approved by Monash University’s Human Research Ethics Committee (MUHREC) [project number: 11275].

### Statewide survey methods

A survey was employed for generating the long list due to its ability to capture responses from a large and diverse sample. The primary target audience for the survey was Victorian healthcare consumers. The secondary audience was those who work in the healthcare sector including hospital and health services, legislators and clinicians (“providers”). The survey was designed collaboratively by SCV and BWA.

The survey was piloted with 14 respondents in a focus-group format, facilitated by BWA researchers. As with all project phases, emphasis in recruiting for piloting was consistent with the PROGRESS-PLUS framework, which identifies factors associated with health opportunities and outcomes, specifically place of residence, race/ethnicity/culture/language, occupation, gender/sex, religion, education, socioeconomic status and social capital [20].

Pilot participants were recruited by SCV and purposively sampled to represent consumers, consumer advocacy organizations, researchers in consumer-led policy and practice, and policy-makers. The aim of the pilot was to gather information on question clarity and purpose as well as overarching considerations such as survey length/respondent burden and format. After reviewing the pilot feedback, several changes were made, primarily around question wording, simplification of language, and adopting inclusive language to capture diverse demographic groups—the final version of the survey is provided in Additional file 1: Appendix 1.

The survey was designed to collect data across three key areas:

1. Participant demographics and relationship with the health system (21 questions)
2. Open-text questions designed to explore priorities within the five domains of the PiH framework, framed as suggestions for improving Victorian hospitals without explicitly referencing the framework itself (see Table 1)
3. A list of 12 suggestions for improving healthcare in hospitals, drawn from the pilot phase (e.g. “Make health information easy to access”, “Inform patients of their different healthcare options”). Respondents were asked to rank their top three preferences. The purpose of this pre-prepared list was to capture priorities from those who could not or did not respond to the open-text questions. The list was placed after the open-text items in order not to bias responses to these.

**Table 1:** Survey questions used to capture priorities within the PiH domains

Domain	Survey question
Person- and family-centred services, care and outcomes	What could be done differently in hospitals to ensure that patients are treated as a whole person?
Teams, partnerships, knowledge and shared learnings	How could hospitals support patients and staff to work together for better care?
Participation and shared decision-making	What needs to change for patients and the people who support them to be more involved in healthcare decisions in hospitals?
Equity, diversity, inclusion and responsiveness	What could be done differently to respond to people’s individual needs in hospital?
Health literacy, information and communication	How can hospital staff help patients better understand health information?

The survey was administered using Engage Victoria, an online government consultation platform designed for community input into issues in Victoria [21]. A link on the platform directed participants to an online survey administered using Qualtrics [22]. A paper version of the survey was also made available upon request.

A communication strategy to reach a broad spectrum of consumers and groups was implemented. In addition to substantial social media outreach via Twitter and Facebook, this involved convenience sampling (where individuals are invited to participate because of their accessibility [23]), primarily through SCV networks, which included employees from the Department of Health and Human Services, the Australian Nursing and Midwifery Federation, Transgender Victoria, Victorian Council of Social Service, Youth Affairs Council Victoria, the Health Issues Centre and the Centre for Culture, Ethnicity and Health. Consumers were also recruited to participate in the survey through Victorian health services via community advisory, volunteer and other community engagement structures. Ultimately, over 180,000 people were reached via social media and other online networks with key messages tailored to specific groups; this generated over 3145 visits to the Engage Victoria consultation web page. The survey was in field from 17 November 2017 to 15 January 2018.

Responses to each open-text question were coded by two independent coders. Thematic categories were identified using a “bottom up” approach—starting with the verbatim comments and using common words or phrases to form initial code frames which were revised and grouped into thematically similar categories [24]. Categories were developed iteratively for each question based on the content of the comments and were quality checked by two independent researchers to ensure consistency in categorization. Effort was made when labelling the categories to draw upon the language and words used by respondents. Not all categories were conceptually equivalent, as some participants responded with high-level suggestions (e.g. “more staff”), while others provided more detailed suggestions (e.g. “provide all patients with a case manager”). Frequent similar suggestions warranted their own category; infrequent suggestions were grouped into appropriate high-level categories [25]. The final themes identified reflect the responses as a whole, providing a descriptive overview of the participant responses [26]. All other survey data was analysed using appropriate descriptive statistics.

### Summit methods

A day-long prioritization summit was held to prioritize the suggestions from the categories generated by the survey respondents to a set of five areas for further focus and activity. All summit methods were based upon successful prioritization forums previously conducted by the research team [15–19]. The lead facilitator (PB) and scribe (BW) were from BWA, and summit participants were identified and invited by SCV.

Due to the overrepresentation of health professionals in the survey (see “Results” section below), the summit was designed to have majority consumer representation—achieving a ratio of 2:1 consumers to health professionals. Purposeful sampling was employed with individuals identified through SCV networks and contacts against the following criteria to ensure equity, inclusion and representation of diverse consumer groups and key service providers. A stakeholder matrix was used to identify and invite participants representing the following groups:

- *Consumers:* Those representing and/or with experience of Aboriginal and Torres Strait Islander (ATSI); lesbian, gay, bisexual, transgender, queer and intersex (LGBTQI); culturally and linguistically diverse (CALD); disability; mental illness; living in regional, rural areas; family violence or homelessness; young and older age; carers, people; different health settings and a range of health conditions; males. Considerable efforts were made to remove barriers to participation in the workshop, including the provision of accessible information before and after the workshop and provision of physical and/or cognitive assistance to facilitate participation in the workshop.
- *Health service providers:* Those representing and/or with experience of disability, ATSI, LGBTQI, consumers organizations, quality managers, health services (metropolitan, regional and rural) and relevant academic research areas.

Prior to the summit, participants were sent an ethics-approved explanatory statement and consent form; a summit overview containing details of the venue, information about the survey that informed the summit and a broad overview of the agenda; and a PiH framework factsheet. The summit opened with group introductions and opening presentations, during which the facilitator emphasized principles of respectful discussion, the importance of treating the information shared in the room as confidential, and the option of stating something “off the record”. The remainder of the summit focused on the key aim of going from the long list to the short list through three activities:

#### Live survey to identify top 15 priorities

First, a survey was administered using Qualtrics [22]. Each of the long lists (i.e. thematic categories identified in the statewide survey) were presented, and participants were asked to nominate their top three preferences. Prior to surveying, respondents were provided with a paper version of the lists with a detailed description of each category (see Additional file 1: Appendix 2). Time was allowed to examine the lists and ask questions to ensure adequate understanding of the categories. Lists were not sent to participants prior to the summit to avoid inequities from some people examining them in detail and others not engaging with them prior to the day. After the survey, the top three priorities (or four where there were equal votes for a third position) were presented back to the group. The top priorities were identified by gross number of votes, regardless of whether these were first, second or third priority. Priority level was only used to infer the top three where the total number of votes was equal for third and fourth place. These were aggregated and de-duplicated to create a list of the top 15 priorities.

#### Whole-group discussion to generate top five priorities

Next, a facilitated whole-group discussion was conducted to discuss the similarities and overlap between the 15 priorities and, through this, reduce the list to the top five. This number was predetermined by SCV in consultation with the research team and made explicit to summit participants. Consistent with principles of facilitation for diverse groups, care was taken to be respectful of all contributions and display empathy towards descriptions of sensitive issues that often arise when discussing healthcare experiences.

#### Small-group discussion of top five priorities

Finally, facilitated small-group discussions provided participants with the opportunity to discuss one priority issue in more detail, including which populations were important, what needed to change and the key challenges. SCV developed a structured facilitation guide for this discussion and facilitated the five groups. Following the small-group discussion, each group reported back, and notes were taken in real time of the feedback reports by the scribe.

Survey data generated in the summit was analysed using appropriate descriptive statistics. For all other data, a narrative summary based on the scribe and the small-group facilitator’s notes was undertaken. Participants were asked to complete a feedback survey following the summit.

## Results

### Statewide survey results

**Fig. 1:**
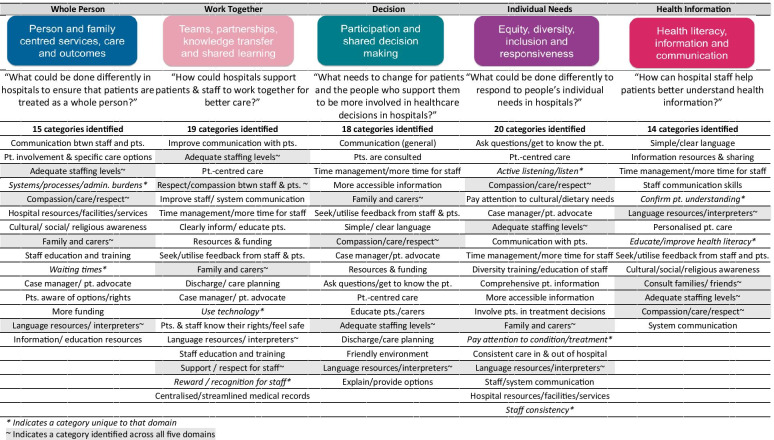
Thematic categories identified across the PiH domains with corresponding survey questions—ordered by frequency of occurrence

The survey had 680 respondents (671 online and 9 paper surveys were completed). The majority of respondents had a Bachelors’ degree or higher (500 respondents; 74%), were employed (520; 77%) and identified as female (505; 74%)—see Additional file 1: Appendix 3, Table A1. Additionally, the respondents comprised 485 providers (71%—those who have worked in healthcare) and 195 consumers (29%—those who have never worked in healthcare)—see Additional file 1: Appendix 3, Table A2. Overall, there was some diversity across the key PROGRESS domains of place of residence (163; 24% outside of capital city); occupation (144; 21% not employed); gender (505; 74% female, 8; 1% self-describe, 5; 1% prefer not to respond); education; and socioeconomic status. However, females, healthcare workers and those with higher education qualifications were over-represented. Figure 1 summarizes the thematic categories identified from the survey (see Additional file 1: Appendix 2 for definitions). In addition, each question included the categories “other” (all 3%), “Not sure/no change necessary” (range: 3–7%) and “No answer” (range: 5–16%). Similar categories were identified across multiple domains in the framework. While the categories are presented in order of frequency for each domain, frequencies were not used to determine priorities or influence prioritization—this was the focus of the summit, during which participants were presented with all categories with no reference to frequencies.

**Fig. 2:**
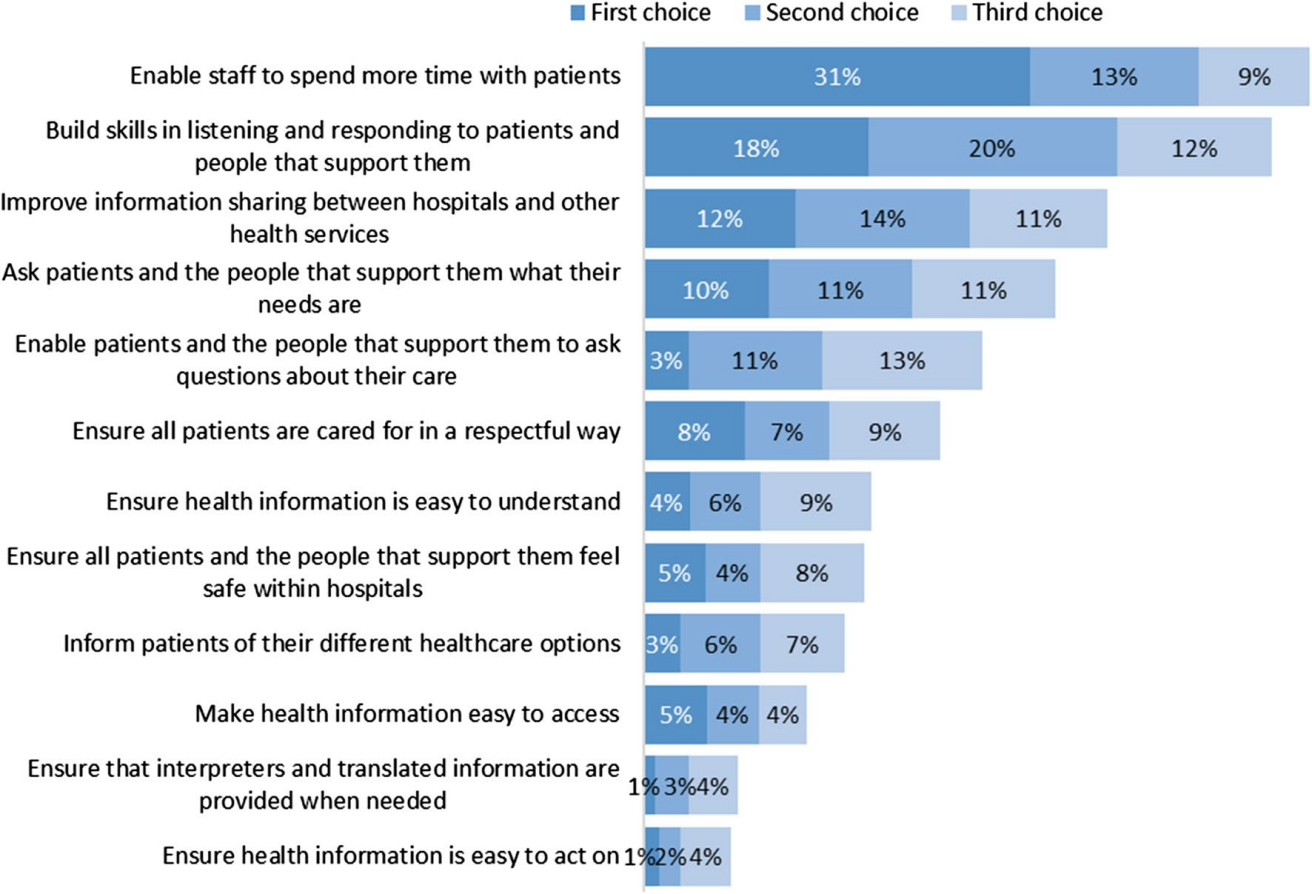
Results of ranking of list of 12 ideas for improving healthcare in hospitals

Figure 2 presents results from the pre-populated list of suggestions in which respondents were asked to rank their top three preferences for improving healthcare in hospitals.

The most frequent suggestions for improving healthcare in Victorian hospitals did not vary substantially across the five domains of the PiH framework, or between the open-text and ranking questions of the survey. For example, there were suggestions across all five domains relating to staff numbers and the time staff spent with patients, respect and compassion, provision of language resources, and involving family members and carers in patient care. Furthermore, every domain included a category relating to communication. Similarly, the highest-ranked items from the pre-populated list were “enable staff to spend more time with patients”, “build skills in listening and responding to patients and people that support them” and “improve information sharing between hospitals and other health services” (see Fig. 2).

### Summit results

The summit was attended by 31 people—21 consumer representatives and 10 people representing the healthcare sector. The summit participants included at least one representative from each of the areas identified in the purposeful sampling matrix, with some attendees representing more than one target group.

**Fig. 3:**
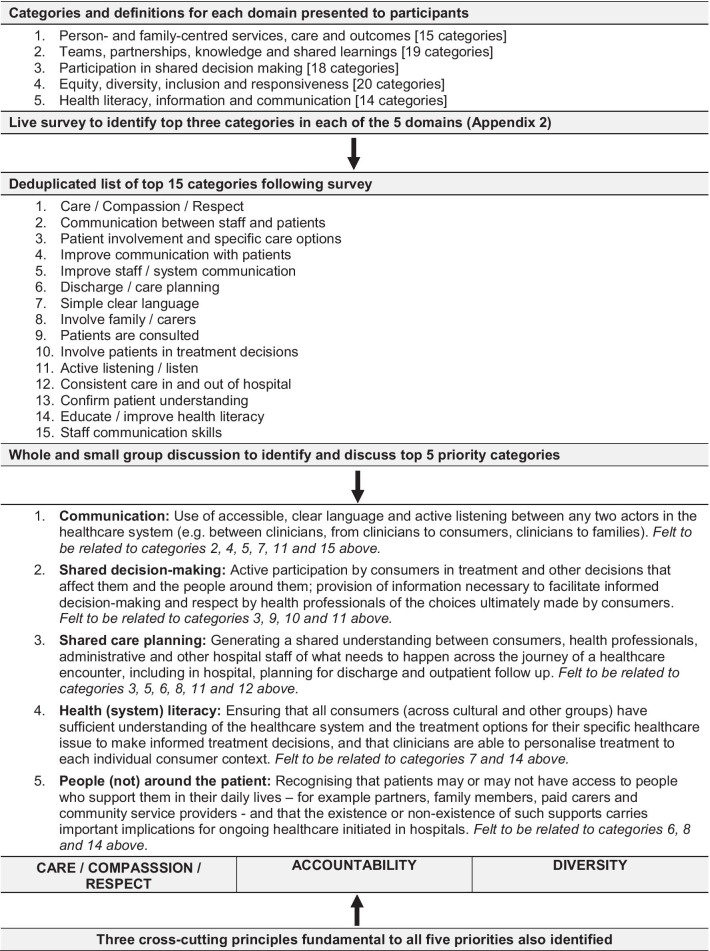
Summary of summit outcomes

Results of the summit are summarized in Fig. 3.

The live survey identified 15 categories (the short list) for further discussion. During the large group discussion, it was recognized that there were a number of conceptually similar categories within the list (e.g. “communication between staff and patients” and “improve communication with patients”) that could be merged under broader topic headings. It was recognized that this would lose some detail regarding individual priorities, but it was important for generating a manageable number of priorities for further development.

During the large group discussion, five priorities were identified and defined—communication, shared decision-making, shared care planning, health (system) literacy and people (not) around the patient. The definition of each category, generated through discussion, focused on specific, practical descriptions of what each of the priorities would mean in terms of observable behaviours and processes (Fig. 3). The discussion also identified three cross-cutting principles that were thought to be fundamental to each of the top five priorities and therefore, not divisible within these—care/compassion/respect, accountability and diversity. Collectively, the priority categories encompassed 13 of the 15 highly ranked categories from the live summit survey—one of the categories (care/compassion/respect) became a cross-cutting principle, and another category (confirm patient understanding) was not thought to directly relate to the five priority categories or represent an overarching principle.

Small-group discussions further unpacked and detailed the five priority areas, with small-group discussions fed back to the large group. Details of these discussions are contained in Additional file 1: Appendix 4. These discussions were shorter than planned, as the large group discussion was more extensive than anticipated. However, it was recognized that the small-group discussion could not meaningfully proceed without identifying the top five priorities.

#### Participant feedback from summit

Participant feedback was collected in real time during the summit—participants were encouraged to ask questions, clarify issues or comment on outcomes of discussion, with responses given by the facilitator—as well as through a feedback form at the end of the day. Twenty-six people completed the feedback survey (response rate 84%), and feedback was generally positive, with 21/26 agreeing with the statement “The information I received before coming helped me understand what was expected of me before I came” (5 neutral/0 disagree) and 25/26 agreeing with the statements “The materials and resources used during the day helped me understand my role and make a contribution” and “I felt that my contribution was heard and valued” (1 neutral/0 disagree). There were almost twice as many responses to the open-ended question “What do you feel worked well today?” (*n* = 47 responses) than responses to the question “What do you feel could have been improved?” (*n* = 24). Some participants felt that the break-out discussions were too short. The top five priorities are all complex and could easily generate a day (or more) of discussion and debate. We had to balance depth of discussion against the primary objective of generating a shared understanding of a small number of priorities that can feasibly be the focus of SCV policy implementation.

Responses to the final open-ended question “Do you have any other thoughts, comment, reflections or ideas?” are presented in Additional file 1: Appendix 5.

Consumers have been central to the PiH framework from the concept design phase preceding this project to the prioritization of themes/domains presented in this paper. To maintain this engagement in post-summit implementation, consumers were engaged via a series of focused discussions in shaping the consumer priorities in each domain. All Victorian health services are required to commit annually to improvement priorities against at least two PiH framework domains. At a health service level, consumers have helped to shape the statements of intent pertaining to this requirement. A follow-up PiH outcomes summit to review progress and share insights and outcomes was planned; however, this has been delayed until 2022 due to the COVID-19 pandemic.

## Discussion

This project aimed to identify priorities for improvement relating to a proposed PiH policy framework from the perspective of Victorian healthcare consumers and those who work in the healthcare sector. Close collaboration between prioritization researchers and policy-makers based on established principles of prioritization was used to address this aim. A statewide survey of Victorians representing key stakeholders generated between 14 and 20 thematic categories across the five domains of the proposed framework. Identified priorities represented consistent themes across domains related to communication, staff time with patients, respect and compassion, language resources, and involving family and carers in patient care. A subsequent in-person summit identified and defined five high-level priorities from the thematic categories identified in the survey—communication, shared decision-making, (shared) care planning, health (system) literacy and people (not) around the patient; underpinned by three cross-cutting principles—care/compassion/respect, accountability and diversity.

A key strength of this work was the connection between prioritization theory and real-world policy implementation. Collaboration between academia and government is mutually beneficial and consistent with evidence-based recommendations to make research relevant, understand policy processes and be accessible to policy-makers [27, 28]. While recruitment was still challenging, SCV’s strong consumer focus gave the research team access to a rich network of consumer organizations and individuals and the necessary mechanisms to access them—in particular through the established web-based Engage Victoria consultation platform and the targeted invitations for the summit. In preparation for the summit, care was taken to personalize communication and ensure that potential barriers to participation—including transport, remuneration, child care and specific physical and cognitive disability access requirements—were identified and addressed. The success of this high-intensity engagement (which involved approximately 6 to 8 contact points between SCV and each of the summit participants) is reflected by the positive participant feedback from the day. In addition to the inherent strength of the academic–government partnership model of this project, each party brought substantial resources and expertise to the project. The research team (BWA) had considerable experience of prioritization theory and processes, as reflected by a number of previously published and successful prioritization projects [15–19], while the government agency (SCV) had spent several years researching and developing the PiH framework, and was committed to partnering with consumers, including through the appointment of a consumer lead as part of the PiH implementation team. SCV therefore brought deep knowledge of its origin, aim and implementation to the partnership.

The study also had several limitations. First, surveys are subject to a range of general limitations, such as recall errors, social desirability bias [29] and language barriers (the survey was only administered in English). Despite these limitations, a survey was the most appropriate and feasible method of generating a long list. Second, the survey sample was not balanced, with a notable overrepresentation of respondents who had worked in healthcare. Furthermore, a response rate could not be generated owing to the convenience distribution method. Although our analysis did not reveal substantial differences between demographic and healthcare experience groups, these limitations are possible sources of bias. Third, during the summit, it was difficult to balance depth of discussion against the primary objective of generating a shared understanding of a small number of priorities that can feasibly be the focus of SCV policy implementation. The top five priorities are all complex and could each have generated a day (or more) of small-group discussion and debate. Some participants felt that the small-group discussions could have been longer. Although the feedback from the day was largely positive, it is always important to carefully consider the optimal balance between various activities in any day-long event such as this. It is never possible to achieve a balance between multiple tasks in a day-long workshop that satisfies all participants. In our experience, focusing on a small number of tasks that are relatively short is preferable to framing an ambitious agenda of in-depth discussion that is not achieved, leading to dissatisfaction at the day's end. Finally, in any prioritization project, it is important to recognize that the most frequently identified suggestions represent those issues which are top of mind at a point in time. The question of whether frequently identified data in qualitative research are therefore “the most important” is a vexed one and, although beyond the scope of this project, is worthy of reflection.

There are relatively few studies seeking consumer input into health policy implementation, when compared to the volume of literature on patient input into health research, for example through the Patient-Centered Outcomes Research Institute (PCORI) [30] and the James Lind Alliance [1]. This has led to calls for better understanding and integration of theoretical priority-setting approaches to real-world political institutions; [31, 32] less tokenistic, more substantive decision-making processes; and greater consideration of equality and diversity in patient and public involvement in healthcare quality improvement [33]. A scoping review of public and vulnerable populations’ participation in health system priority-setting published in 2019 found that of 96 included papers, only 24 mentioned public participation in priority-setting, and only six reported on participation of vulnerable populations [34]. These examples were focused on methodologies/approaches or specific health conditions and therefore not wholly comparable to the scope of this study. Addressing these recommendations and gaps in the literature is a major strength of this project. Comparison of the findings of this study with our own previous research (focused on research prioritization) does reveal some consistencies in findings. For example, a summit to identify high-priority research questions for systematic reviews in osteoarthritis conducted in Singapore with 29 participants (including 20 consumers) identified “increase the level and quality of communication” as the equal highest-ranked priority [35]. A separate review prioritization project with a similar approach (large-scale survey followed by summit) identified communication, patient/family involvement, consumer engagement and patient-centred care as the highest priorities [15, 36].

There was overlap between domains and categories at multiple levels of this project—from the initial five PiH domains in the proposed framework and the thematic categories identified in survey data analysis to the 15 highly ranked categories from the summit survey—many of which ultimately ended up being merged during the summit discussions to create and define the top five stakeholder priorities. These observations can be interpreted in several ways. First, they confirm that there is conceptual overlap between domains. Second, they imply that person- and family-centred services are viewed as critical in the eyes of the project participants. Finally, in addition to refining the five domains, the prioritization activities demonstrate that the domains in the proposed framework (themselves informed by previous literature) have face validity to the audience they seek to serve, as the concerns and preferences of consumers speak strongly to them.

Based on the outcomes of this research, the PiH Framework was refined by SCV and launched in 2019, replacing the “Doing it with us not for us” framework [7] and the Cultural Responsiveness framework [8]. The prioritization methodology and codesign approach resulted in a coherent consumer participation framework which helped bring consistency to participation in one’s healthcare; aided health services making efforts to involve consumers to deliver care that is safe, effective, person- and family-centred, equitable and clinically effective; and clearly described consumer priorities. The final framework comprised the following domains:

1. *Personalized and holistic:* Individualized and connected care; compassion and respect
2. *Working together:* Codesign of care with patients, families and clinicians; coordination and continuity of care
3. *Shared decision-making:* Use of decision aids, decision support coaching
4. *Equity and inclusion:* Patient-reported language services provision; cultural safety, diversity of consumer participation
5. *Effective communication:* Respectful communication, health literacy.

Implementation has been a major focus since the framework was finalized. A self-assessment tool developed by SCV has since been used to guide all Victoria’s health services in identifying focus domains and priorities (which may align to the service’s existing activities and goals). Each health service is required to complete a statement of intent committing to action in these areas. Effective communication was the highest priority domain (46), followed by working together (39), equity and inclusion (33), personalized and holistic (21) and shared decision-making (21). SCV is also currently exploring how best to measure and evaluate the achievements of PiH.

The cross-cutting concepts of “care/compassion/respect”, “accountability” and “diversity” could be integrated into monitoring and evaluation of actions that health services commit to. Examples of evaluation questions are:

1. What measures are in place to ensure that care, compassion and respect for consumers are shown in implementing proposed actions (e.g. through consumer feedback)?
2. Are proposed actions auditable so that the level of implementation into practice can be determined and fed back to those accountable?
3. Have cultural, gender and other types of diversity been adequately considered in the development of systems, supports and materials to implement proposed actions?

A number of questions remain unanswered at the conclusion of this project. As Victorian health services are only at the beginning of implementation, it is not known to what extent the framework and the priorities are tractable and feasible to implement, and to what extent the activities committed to address the needs articulated by this process—a research gap identified by other authors in this field [3]. Further surveys, monitoring and evaluation and/or a follow-up summit would be required to answer this question. As this is one of few projects connecting consumer priorities to health policy implementation, there is a dearth of comparable studies outside of research prioritization. Adoption of a similar approach in other Australian and international jurisdictions, combined with long-term evaluation, would ultimately facilitate the creation of more robust review-level evidence in the critical area of consumer-informed health policy implementation.

## Conclusions

This collaborative project between Monash University’s BehaviourWorks Australia and the Victorian Government’s Department of Health and Human Services aimed to identify priorities for implementation of a PiH framework from the perspective of healthcare consumers and other relevant groups. Based on principles of codesign and using extensive consumer and other networks, a statewide survey of 680 Victorians was undertaken which identified key themes across the five domains of the framework relating to communication, staff time with patients, respect and compassion, language resources and involving family and carers in patient care. A subsequent day-long summit was attended by 31 participants including 21 consumer representatives. The summit identified and defined five high-level priorities from the thematic categories identified in the survey—communication, shared decision-making, (shared) care planning, health (system) literacy and people (not) around the patient, underpinned by three cross-cutting principles—care/compassion/respect, accountability and diversity.

This study demonstrates the value of coproduction of research between government and academia, which enabled the research team to harness the deep healthcare consumer networks of the partnering organization. The input of healthcare consumers has enabled design, implementation and ongoing monitoring of the PiH framework to be grounded in the experiences of the people that it seeks to serve.

## Supplementary Information

**Additional file 1. Appendix 1.** Partnering in Healthcare Survey. **Appendix 2.** Thematic analysis category descriptions as provided to summit participants; results of live survey in summit to identify top three categories. **Appendix 3**. Survey respondent profile. **Appendix 4.** Key points from small-group discussion. **Appendix 5.** Responses to “Do you have any other thoughts, comment, reflections or ideas?” from the summit participant feedback survey.

## Data Availability

The data sets used and/or analysed during the current study are available from the corresponding author on reasonable request.

## Abbreviations

ATSI: Aboriginal and Torres Strait Islander
BWA: BehaviourWorks Australia
CALD: Culturally and linguistically diverse
LGBTQI: Lesbian, gay, bisexual, transgender, queer and intersex
PiH: Partnering in Healthcare
SCV: Safer Care Victoria
