# Supplementary material for: From policy to practice: prioritizing person-centred healthcare actions in the state of Victoria
Source: Health Res Policy Syst. 2021 Oct 26;19:133. doi: 10.1186/s12961-021-00782-2 (PMC8546749; doi:10.1186/s12961-021-00782-2)
Supplement: Supplementary file 1 — Additional file 1. Appendix 1. Partnering in Healthcare Survey. Appendix 2. Thematic analysis category descriptions as provided to summit participants; results of live survey in summit to identify top three categories. Appendix 3. Survey respondent profile. Appendix 4. Key points from small-group discussion. Appendix 5. Responses to “Do you have any other thoughts, comment, reflections or ideas?” from the summit participant feedback survey. [file 12961_2021_782_MOESM1_ESM.docx]

# Appendix 1: Partnering in Healthcare Survey

**INTRODUCTION & SCREENING**

Welcome to the Partnering in Healthcare survey. Before continuing, please read the below information carefully.

This survey is being funded by Safer Care Victoria at the Department of Health and Human Services and is being conducted by Monash University. The aim of the survey is to identify ways to improve healthcare experiences and outcomes across the Victorian health system. We are interested in hearing from patients, families, healthcare providers and health services. The responses collected in this survey will be used to develop programs and initiatives to improve healthcare for all Victorians in the future.

If you agree to participate, we will only need about 15 minutes of your time. There should be no inconvenience or discomfort for you, other than the time taken to complete the survey. Your participation is completely voluntary. Even if you agree to participate, you can choose to skip any question you do not wish to answer or can withdraw from the survey any time before you submit your responses. If you withdraw, any answers you provided will not be used. If you participate, you will not be asked for your name or contact details, however we are interested in certain health and personal information. Your answers will only be accessible to the research team at Monash and the Department of Health and Human Services. Your answers will be collected and managed in line with the *Privacy and Data Protection Act 2014* and Department of Health and Human Services Privacy Policy.

Upon completion of the research, a report will be provided to Safer Care Victoria in summary format so that no individual can be identified. The research findings may also be submitted for publication or used in conference presentations (also in summary format). Data files will be held for a minimum of five years, secured on the Monash University network with restricted access, limited to the research staff only. The data may be retained and used by the research team for comparative purposes in the future.

If you have any concerns or complaints about the project, you can contact the Executive Officer of the Monash University Human Research Ethics Committee. Project number: 11275

Executive Officer

Monash University Human Research Ethics Committee (MUHREC)

Room 111, Building 3e

Research Office

Monash University VIC 3800

Tel: +61 3 9905 2052

Email: [muhrec@monash.edu](mailto:muhrec@monash.edu)

First we have a few questions about you. We are collecting this information to ensure we speak with a range of different people.

**D1: What is your gender?**

- Female
- Male
- Self-describe _________________
- Prefer not to respond

**D2: What is your age?**

___ ___ years

**D3: What is your postcode?**

___ ___ ___ ___

**MC. Have you ever worked in the healthcare sector?**This could be as a clinician, manager, policy maker, administrator, government employee, researcher, or something else.

- Yes (please specify)________________________
- No

We’d also like to understand a bit about your health so we have a better idea of your experiences with the healthcare services.

Your responses will be completely anonymous and you can skip any question that you are not comfortable answering.

**D17. Do you identify as a person with disability?**

- Yes
- No
- Prefer not to respond

**D18. Do you currently have any health conditions?**

- Yes – acute / short term (please specify) ___________________________________________
- Yes – ongoing (please specify) _________________________________________
- No
- Prefer not to respond

**D19. Do you support someone who has a long-term health condition or disability, or is elderly?**

- Yes (please specify) ________________________________
- No
- Prefer not to respond.

**D20. How often do you use hospital services?**

- More than once a week
- At least once a week
- At least once a month
- At least once every six months
- At least once a year
- Less than once a year

**SECTION A: SUGGESTIONS FOR IMPROVING HEALTHCARE**

Next, we would like to ask some questions about how healthcare at hospitals can be improved.

In your responses, think about what you would do differently **if you were in charge of Victorian hospitals.**

Please be as descriptive as possible in your answers. Remember, there are no right or wrong answers, we want your opinion, and you can skip any question you do not wish to answer.

***PF1. What could be done differently in hospitals to ensure that patients are treated as a whole person?***

***PC1. How could hospitals support patients & staff to work together for better care?***

***SD1. What needs to change for patients and the people who support them to be more involved in healthcare decisions in hospitals?***

***EC1. What could be done differently to respond to people’s individual needs in hospital?***

***HL1. How can hospital staff help patients better understand health information?***

**V1. Overall, if you could change one thing about the way healthcare is provided in hospitals in Victoria, what would it be and why?**

**SECTION B: PRIORITIES FOR IMPROVING HEALTHCARE**

Below is a list of suggestions that could improve healthcare in hospitals.

**PR1. Based on the items below, please rank the THREE suggestions you think would be most helpful for improving healthcare in Victorian hospitals?**

**Place a 1 next to your first preference, a 2 next to your second preference and a 3 next to your third preference.**

| 1. Make health information easy to access |  |
| --- | --- |
| 1. Enable staff to spend with more time with patients |  |
| 1. Build skills in listening and responding to patients and people that support them |  |
| 1. Improve information sharing between hospitals and other health services |  |
| 1. Enable patients and the people that support them to ask questions about their care |  |
| 1. Inform patients of their different healthcare options |  |
| 1. Ask patients and the people that support them what their needs are |  |
| 1. Ensure that interpreters and translated information are provided when needed |  |
| 1. Ensure health information is easy to understand |  |
| 1. Ensure health information is easy to act on |  |
| 1. Ensure all patients are cared for in a respectful way |  |
| 1. Ensure all patients and the people that support them feel safe within hospitals |  |

**SECTION C: DEMOGRAPHICS**

Finally, we have a few questions about you. We are collecting this information to better understand who is completing the survey and to find out if different people have different suggestions for improving healthcare in Victorian hospitals. You can skip any question you do not wish to answer.

**D4: Do you identify as Aboriginal or Torres Strait Islander?**

- Yes
- No
- Prefer not to respond

**D5: Besides English, what is the main language you speak at home?**

- Only speak English
- Mandarin
- Italian
- Greek
- Vietnamese
- Arabic
- Cantonese
- Punjabi
- Hindi
- Filipino/Tagalog
- Persian/Dari
- Other (please specify) ______________________________
- Prefer not to respond

**D6: In which country were you born?**

- Australia
- United Kingdom
- India
- China
- New Zealand
- Vietnam
- Italy
- Sri Lanka
- Philippines
- Malaysia
- Greece
- Other (please specify) ______________________________
- Prefer not to respond

**D7: Which of the following best describes your sexual orientation?**

- Heterosexual
- Homosexual (gay or lesbian)
- Bisexual
- Asexual
- Another sexual orientation (please specify) ______________________________
- Prefer to not respond

**D8: Do you identify as trans and gender diverse?**

- Yes
- No
- Self-describe ______________________________
- Prefer not to respond

**D9: Intersex is a general term for a broad range of physical, hormonal or genetic characteristics or variations that lie between medical or social norms of female or male.**

**Are you intersex or do you have experience of the above?**

- Yes
- No
- Self-describe _______________________________
- Prefer not to respond

**D10: What is the highest level of education qualification you have completed?**

- Year 10 or below
- Year 11
- Year 12
- Certificate I/II
- Certificate III/IV
- Diploma/Advanced Diploma
- Bachelor’s degree
- Graduate diploma/Graduate certificate
- Postgraduate degree
- Prefer not to respond

**D11: What is your current employment status?**(If you are employed but are currently on leave, this would still be classified as employed)

- Employed full time
- Employed part time
- Employed casually
- Student only **🡪 GO TO D14**
- Engaged in home duties or volunteer work **🡪 GO TO D14**
- Retired **🡪 GO TO D14**
- Unemployed but currently seeking employment **🡪 GO TO D14**
- Unemployed and not seeking employment **🡪 GO TO D14**
- Prefer not to respond **🡪 GO TO D14**

**D12: Which of the following best describes your main occupation?**

- **Managers and administrators**

Chief executives, General Managers and Legislators, Farmers and farm managers, Specialist managers, Hospitality, retail and service managers

- **Professionals & Associate professionals**

Arts and Media Professionals, Business, Human Resource and Marketing Professionals, Design, Engineering, Science and Transport Professionals, Education Professionals, Health Professionals, ICT Professionals, Legal, Social and Welfare Professionals

- **Technicians and trade workers**

Engineering, ICT and Science Technicians, Automotive and Engineering Trades Workers, Construction Trades Workers, Electrotechnology and Telecommunications Trades Workers, Food Trades Workers, Skilled Animal and Horticultural Workers, Other Technicians and Trades Workers

- **Community and personal service workers**

Health and Welfare Support Workers, Carers and Aides, Hospitality Workers, Protective Service Workers, Sports and Personal Service Workers

- **Clerical and administrative workers**

Office Managers and Program Administrators, Personal Assistants and Secretaries, General Clerical Workers, Inquiry Clerks and Receptionists, Numerical Clerks, Clerical and Office Support Workers, Other Clerical and Administrative Workers

- **Sales workers**

Sales Representatives and Agents, Sales Assistants and Salespersons, Sales Support Workers

- **Machinery operators and drivers**

Machine and Stationary Plant Operators, Mobile Plant Operators, Road and Rail Drivers, Storepersons

- **Labourers and related workers**

Cleaners and Laundry Workers, Construction and Mining Labourers, Factory Process Workers, Farm, Forestry and Garden Workers, Food Preparation Assistants, Other Labourers

- **Something else (please describe) _____________________________________**

**D13. Which of the following best describes the business or service carried out by your employer at the place where you work?**

- Agriculture, forestry and fishing
- Mining
- Manufacturing
- Electricity, gas, water and waste services
- Construction
- Wholesale trade
- Retail trade
- Accommodation and food services
- Transport, postal and warehousing
- Information media and telecommunications
- Financial and insurance services
- Rental, hiring and real estate services
- Professional, scientific and technical services
- Administrative and support services
- Public administration and safety
- Education and training
- Health care and social assistance
- Arts and recreational services
- Other services (please describe) _______________________

**D14. Which of the following categories best describes your household?**

- Person living alone **🡪 GO TO D16**
- Couple with no children **🡪 GO TO D16**
- Couple with a child or children at home
- Couple whose children have all left home **🡪 GO TO D16**
- Single parent with a child or children at home
- Single parent whose children have all left home **🡪 GO TO D16**
- Non-related adults sharing a house/apartment/flat**🡪 GO TO D16**
- Other type of household (please specify) ________________________ **🡪 GO TO D16**
- Prefer not to respond **🡪 GO TO D16**

**D15. You mentioned that you have children living with you at home. How many children do you have at home in the following age brackets?**

- 0-2 years: ___ ___
- 3-5 years: ___ ___
- 6-10 years: ___ ___
- 11-15 years: ___ ___
- 16-17 years: ___ ___
- 18 years or older ___ ___

**D16. What is your approximate HOUSEHOLD income?**(This includes income from wages and salaries, government benefits, pensions, allowances and any other income you usually receive, before deductions for tax, superannuation contributions, health insurance, amounts salary sacrificed, or any other automatic deductions.)

- $1-$199 per week ($1-$10,399 per year)
- $200-$299 per week ($10,400-$15,599 per year)
- $300-$399 per week ($15,600-$20,799 per year)
- $400-$599 per week ($20,800-$31,199 per year)
- $600-$799 per week ($31,200-$41,599 per year)
- $800-$999 per week ($41,600-$51,999 per year)
- $1,000-$1,249 per week ($52,000-$64,999 per year)
- $1,250-$1,499 per week ($65,000-$77,999 per year)
- $1,500-$1,999 per week ($78,000-$103,999 per year)
- $2,000 $2,499 per week ($104,000-$129,999 per year)
- $2,500-$2,999 per week ($130,000-$155,999 per year)
- $3,000-$3,499 per week ($156,000-$181,999 per year)
- $3,500-$3,999 per week ($182,000-$207,999 per year)
- $4,000-$4,999 per week ($208,000-$259,999 per year)
- $5,000 or more per week ($260,000 or more per year)
- Negative income
- Nil income
- Prefer not to respond

**END & THANK YOU**

Thank you for participating in this important research.

That's the end of the survey questions.

If you would like to know more about the survey you can contact:

Safer Care Victoria

Lidia Horvat

Tel: +61 3 9096 9008

Email: [lidia.horvat@dhhs.vic.gov.au](mailto:lidia.horvat@dhhs.vic.gov.au)

Monash University

Kim Borg

Tel: +61 3 9902 0351

Email: [kim.borg@monash.edu](mailto:kim.borg@monash.edu)

If you have any concerns or complaints about the project, you can contact:

Project number: 11275

Executive Officer

Monash University Human Research
Ethics Committee (MUHREC)

Room 111, Building 3e

Research Office

Monash University VIC 3800

Tel: +61 3 9905 2052

Email: [muhrec@monash.edu](mailto:muhrec@monash.edu)

If you have feedback relating to a Victorian public health service, you can contact the patient representative or consumer liaison officer at the relevant hospital. To find hospital contact information, see list of [Public hospital websites in Victoria](https://www2.health.vic.gov.au/hospitals-and-health-services/public-hospitals-victoria).

If you want more information regarding the Department of Health and Human Services Privacy Policy, please see the linked [policy](https://dhhs.vic.gov.au/publications/privacy-policy).

If you experienced any distress while completing this survey and would like immediate support, please contact Beyondblue on **1300 22 4636.**

# Appendix 2: Thematic analysis category descriptions as provided to Summit participants; results of live survey in summit to identify top 3 categories

*Person- and family-centred services, care and outcomes*

| **CATEGORY LABEL** | **DESCRIPTION** |
| --- | --- |
| Case manager/ patient advocate | Provide patients with a support person at the hospital such as a case manager or patient advocate. Conduct community consultation and include reps on boards. |
| Cultural/ social/ religious awareness | Increased awareness of and services/facilities for individual's cultural, religious and social preferences. |
| Language resources/ interpreters | Provide non-English speaking patients with appropriate resources including information in their preferred language and access to interpreters. |
| Patient involvement and specific care options | Encourage collaboration between patients, their families, and staff and respond to individual care preferences. |
| Adequate staffing levels | Ensure enough staff are available. This may include staff generally, or specific references to nurses, specialists, etc. It also covers nurse to patient ratios, getting the 'right' staff, and giving staff more time to spend with patients. |
| Staff education and training | Educate and inform hospital staff generally and specifically. This includes better understanding of patients and better training in specific areas such as mental health. |
| Patients aware of options/ rights | Provide sufficient information to patients and ensure they have the opportunity to ask questions about their rights and treatment/care options. |
| Compassion/ care/ respect | Create an environment which is respectful, safe and positive for patients and their families. This includes being friendly, respecting patient's decisions, being treated as equals. |
| Waiting times | Avoid long waiting periods for patients - particularly in relation to appointments made with specialists. |
| Information/ education resources | Ensure information and educational resources are easily available for patients and their families. |
| Communication between staff and patients | Improve the communication process between staff and patients including sharing information and listening to each other. |
| Family and carers | Involve patient's families and carers in discussions and decisions, particularly when the patient is vulnerable or has language or cognitive difficulties. |
| Systems/ processes/ administrative burdens | Changes to the healthcare system including sharing of information, use of technology, data, processes, paperwork and 'red tape'. |
| Hospital resources/ facilities/ services | General suggestions to improve hospital facilities and services such as better food, more beds, and cheaper parking. |
| More funding | Provide more funding to hospitals or increased funding for specific services. |

*Top 3 priorities from live survey at summit*

| Compassion / care / respect | 17 votes |
| --- | --- |
| Communication between staff and patients | 14 votes |
| Patient involvement and specific care options | 12 votes |

*Teams, partnerships, knowledge and shared learnings*

| **CATEGORY LABEL** | **DESCRIPTION** |
| --- | --- |
| Case manager/ patient advocate | Provide patients with a support person at the hospital such as a case manager or patient advocate. Conduct community consultation and include reps on boards. |
| Respect/ compassion between staff and patients | Ensure staff and patients are respectful, compassionate, and sympathetic towards each other. |
| Patient-centred care | Mentions of patient-centred care, includes suggestions for formal training and for improving general understanding. |
| Clearly inform/ educate patients | Provide sufficient information to patients including general education on the healthcare system and managing their health outside of hospital. |
| Adequate staffing levels | Ensure enough staff are available. This may include staff generally, or specific references to nurses, specialists, etc. It also covers nurse to patient ratios, getting the 'right' staff, and giving staff more time to spend with patients. |
| Use technology | Embrace new and existing technology and digital services to streamline healthcare provision. |
| Improve communication with patients | Ensure staff are communicating with patients, particularly around getting to know the patient and including them in decisions. |
| Improve staff/ system communication | Improve communication between staff, departments, and the healthcare system (e.g. hospitals and primary care). |
| Resources & funding | Requests for more funding and resources either generally (e.g. more funding) or requests for specific equipment (e.g. more beds). |
| Discharge/ care planning | Better continuity of care outside of hospital, particularly in relation to discharge planning and follow ups. |
| Family and carers | Involve patient's families and carers in discussions and decisions, particularly when the patient is vulnerable or has language or cognitive difficulties. |
| Language resources/ interpreters | Provide non-English speaking patients with appropriate resources including information in their preferred language and access to interpreters. |
| Time management / more time for staff | Allow more time for staff to spend engaging with patients. Improve time management (e.g. appointments) in the hospital. |
| Seek & utilise feedback from staff and patients | Seek out and act on feedback provided by patients, carers, and staff for improving healthcare. |
| Reward / recognition for staff | Provide rewards, incentives, recognition for staff who adopt desirable patient interaction practices. |
| Patients and staff know their rights / feel safe | Provide information and education for staff and patients on their rights and responsibilities. Includes ensuring the environment is safe. |
| Staff education and training | Educate and inform hospital staff generally and specifically. This includes better understanding of patients and better training in specific areas such as mental health. |
| Support / respect for staff | Provide adequate support for staff and ensure they are treated respectfully by patients and other staff. |
| Centralised/ streamlined medical records | Adopt a standardised health record processes which is centralised and streamlined. |

*Top 3 priorities from live survey at summit*

| Improve communication with patients | 13 votes |
| --- | --- |
| Improve staff / system communication | 11 votes |
| Discharge / care planning | 8 votes |

*Participation in shared decision making*

| **CATEGORY LABEL** | **DESCRIPTION** |
| --- | --- |
| More accessible information | Provide patients with enough information which is easy to understand and access. |
| Simple/ clear language | Avoid complex medical jargon and provide information in simple and clear English. |
| Compassion/ care/ respect | Create an environment which is respectful, safe and positive for patients and their families. This includes being friendly, respecting patient's decisions, being treated as equals. |
| Educate patients/ carers | Provide education to patients and their supports on their rights, the health care system, and tools available. |
| Time management / more time for staff | Allow more time for staff to spend engaging with patients. Improve time management (e.g. appointments) in the hospital. |
| Explain/ provide options | Provide patients with options regarding their care and treatment. |
| Seek & utilise feedback from staff and patients | Seek out and act on feedback provided by patients, carers, and staff for improving healthcare. |
| Resources & funding | Requests for more funding and resources either generally (e.g. more funding) or requests for specific equipment (e.g. more beds). |
| Language resources/ interpreters | Provide non-English speaking patients with appropriate resources including information in their preferred language and access to interpreters. |
| Family and carers | Involve patient's families and carers in discussions and decisions, particularly when the patient is vulnerable or has language or cognitive difficulties. |
| Patients are consulted | Consult patients (and their families) and respond to individual preferences for care. |
| Communication (general) | Improve communication between staff and patients/carers and between departments/the health system more generally. |
| Adequate staffing levels | Ensure enough staff are available. This may include staff generally, or specific references to nurses, specialists, etc. It also covers nurse to patient ratios, getting the 'right' staff, and giving staff more time to spend with patients. |
| Case manager/ patient advocate | Provide patients with a support person at the hospital such as a case manager or patient advocate. Conduct community consultation and include reps on boards. |
| Friendly environment | Create a friendly and welcoming environment, including physical space and interactions with staff. |
| Ask questions / get to know the patient | Encourage staff to ask questions in order to get to know patients better and understand their specific needs. |
| Patient-centred care | Mentions of patient-centred care, includes suggestions for formal training and for improving general understanding. |
| Discharge/ care planning | Better continuity of care outside of hospital, particularly in relation to discharge planning and follow ups. |

*Top 3 priorities from live survey at summit*

| Compassion / care / respect | 10 votes |
| --- | --- |
| Simple clear language | 9 votes |
| Involve family / carers & Patients are consulted | 8 votes (tie for 3^rd^) |

*Equity, diversity, inclusion and responsiveness*

| **CATEGORY LABEL** | **DESCRIPTION** |
| --- | --- |
| Pay attention to condition/ treatment | Greater attention paid by staff to patient's individual healthcare needs, condition, treatment options/preferences. |
| Pay attention to cultural and dietary needs | Greater attention paid by staff to patient's individual's personal needs including cultural, social, and dietary. |
| Adequate staffing levels | Ensure enough staff are available. This may include staff generally, or specific references to nurses, specialists, etc. It also covers nurse to patient ratios, getting the 'right' staff, and giving staff more time to spend with patients. |
| Time management / more time for staff | Allow more time for staff to spend engaging with patients and improvement time management (e.g. appointments) in the hospital. |
| Active listening / listen | Staff need to listen more to patients and practice active listening to ensure they understand what the patient is saying. |
| Patient-centred care | Mentions of patient-centred care, includes suggestions for formal training and for improving general understanding. |
| Ask questions / get to know the patient | Encourage staff to ask questions in order to get to know patients better and understand their specific needs. |
| Case manager/ patient advocate | Provide patients with a support person at the hospital such as a case manager or patient advocate. Conduct community consultation and include reps on boards. |
| More accessible information | Provide patients with enough information which is easy to understand and access. |
| Comprehensive patient information | Conduct/improve admissions surveys to collect sufficient information about the patient's needs and preferences. |
| Compassion/ care/ respect | Create an environment which is respectful, safe and positive for patients and their families. This includes being friendly, respecting patient's decisions, being treated as equals. |
| Communication with patients | Ensure staff are communicating with patients, particularly around getting to know the patient and including them in decisions. |
| Involve patients in treatment decisions | Include patients and their families in decisions regarding their treatment. Ensure patients are provided with options. |
| Staff consistency | Consistency in staff treating and attending patients. |
| Family and carers | Involve patient's families and carers in discussions and decisions, particularly when the patient is vulnerable or has language or cognitive difficulties. |
| Consistent care in & out of hospital | Better coordination between hospitals, specialists and primary health so that patients receive consistent care in and out of hospital. |
| Language resources/ interpreters | Provide non-English speaking patients with appropriate resources including information in their preferred language and access to interpreters. |
| Staff/ system communication | Improve communication between staff, departments, and the healthcare system (e.g. hospitals and primary care). |
| Diversity training/ education of staff | Provide more/better training for staff on diversity such as cultural awareness, mental health, identity, disability, etc. |
| Hospital resources/ facilities/ services | General suggestions to improve hospital facilities and services such as better food, more beds, and cheaper parking. |

*Top 3 priorities from live survey at summit*

| Involve patients in treatment decisions | 12 votes |
| --- | --- |
| Active listening / listen | 11 votes |
| Consistent care in and out of hospital | 8 votes |

*Health literacy, information and communication*

| **CATEGORY LABEL** | **DESCRIPTION** |
| --- | --- |
| Simple/ clear language | Avoid complex medical jargon and provide information in simple and clear English. |
| Language resources/ interpreters | Provide non-English speaking patients with appropriate resources including information in their preferred language and access to interpreters. |
| Time management / more time for staff | Allow more time for staff to spend engaging with patients and improvement time management (e.g. appointments) in the hospital. |
| Confirm patient understanding | Actively ask patients if they have understood information shared with them to confirm comprehension. |
| Staff communication skills | General comments around improving staff communication with patients. Includes training, general references to skills, and specific techniques to employ. |
| Seek & utilise feedback from staff and patients | Seek out and act on feedback provided by patients, carers, and staff for improving healthcare. |
| Adequate staffing levels | Ensure enough staff are available. This may include staff generally, or specific references to nurses, specialists, etc. It also covers nurse to patient ratios, getting the 'right' staff, and giving staff more time to spend with patients. |
| Information resources & sharing | Provide more information resources for patients to review and share existing resources. |
| Cultural/ social/ religious awareness | Increased awareness of and services/facilities for individual's cultural, religious and social preferences. |
| Compassion/ care/ respect | Create an environment which is respectful, safe and positive for patients and their families. This includes being friendly, respecting patient's decisions, being treated as equals. |
| Educate/ improve health literacy | Address health literacy among patients and educate staff on addressing health literacy issues. |
| Personalised patient care | Provide individual, personalised care. This includes understanding patient's individual needs. |
| Consult families/ friends | Respect, support, and engage with patient's families, friends, and support people. |
| System communication | Better communication between the hospital, specialists, and primary health care providers such as GPs. |

*Top 3 priorities from live survey at summit*

| Confirm patient understanding | 11 votes |
| --- | --- |
| Educate / improve health literacy | 11 votes |
| Simple clear language & Staff communication skills | 10 votes (tie for 3^rd^) |

# Appendix 3: Survey respondent profile

**Table A1. Demographic profile of survey respondents**

|  | | **Responding sample** | |
| --- | --- | --- | --- |
|  |  | **(n=680)** | |
|  |  | **n** | **%** |
| **Gender** | Female | 505 | 74% |
|  | Male | 162 | 24% |
|  | Self-describe | 8 | 1% |
|  | Prefer not to respond | 5 | 1% |
| **Age group** | Up to 54 | 391 | 58% |
|  | 55 and older | 289 | 43% |
| **Geography** | Greater capital city | 429 | 63% |
|  | Rest of state | 163 | 24% |
|  | Interstate | 88 | 13% |
| **Aboriginal or Torres Strait Islander** | Yes | 18 | 3% |
|  | No | 645 | 95% |
|  | Prefer not to respond | 17 | 3% |
| **Language** | English only | 586 | 86% |
|  | Language other than English | 66 | 10% |
|  | Prefer not to respond | 28 | 4% |
| **Country of birth** | Australia | 508 | 75% |
|  | Overseas | 153 | 23% |
|  | Prefer not to respond | 19 | 3% |
| **LGBTI** | Yes | 58 | 9% |
|  | No | 554 | 81% |
|  | Prefer not to respond | 68 | 10% |
| **Education** | Less than Bachelor’s degree | 163 | 24% |
|  | Bachelor’s degree or higher | 500 | 74% |
|  | Prefer not to respond | 17 | 3% |
| **Employment status** | Employed | 520 | 76% |
|  | Not working | 144 | 21% |
|  | Prefer not to respond | 16 | 2% |
| **Household type** | Lone person | 95 | 14% |
|  | Couple without children | 233 | 34% |
|  | Parent(s) with children at home | 267 | 39% |
|  | Other type of household | 55 | 8% |
|  | Prefer not to respond | 30 | 4% |
| **Number of dependent children** | 0 | 484 | 71% |
|  | 1 | 73 | 11% |
|  | 2 | 87 | 13% |
|  | 3 or more | 36 | 5% |
| **Household income** | Up to $1,499 per week | 188 | 28% |
|  | $1,500 or more per week | 328 | 49% |
|  | Prefer not to respond | 153 | 23% |

**Table A2. Experience with hospitals and healthcare**

|  | | **Responding sample** | |
| --- | --- | --- | --- |
|  |  | **(n=680)** | |
|  |  | **n** | **%** |
| **Ever worked in healthcare** | Yes | 485 | 71% |
|  | No | 195 | 29% |
| **Person with disability** | Yes | 74 | 11% |
|  | No | 590 | 87% |
|  | Prefer not to respond | 16 | 2% |
| **Current health conditions** | Yes - acute / short term | 30 | 4% |
|  | Yes - ongoing | 303 | 45% |
|  | No | 321 | 47% |
|  | Prefer not to respond | 26 | 4% |
| **Support person** | Yes | 220 | 32% |
|  | No | 447 | 66% |
|  | Prefer not to respond | 13 | 2% |
| **Hospital visitation frequency** | More than once a week | 11 | 2% |
|  | At least once a week | 10 | 1% |
|  | At least once a month | 58 | 9% |
|  | At least once every six months | 99 | 15% |
|  | At least once a year | 87 | 13% |
|  | Less than once a year | 175 | 26% |
|  | Prefer not to respond/skipped | 240 | 35% |
| **Healthcare work experience** | Currently works in healthcare industry | 345 | 51% |
|  | Has worked in healthcare before | 146 | 21% |
|  | Never worked in healthcare | 189 | 28% |

# Appendix 4: Key points from small group discussion

Below are the verbatim summaries of the small group discussions as presented, scribed and projected in real time.

#### Communication

Communication should be embedded as a priority for the organisation.

- It is as important as clinical outcomes
- Should include how they recruit, how skills are evaluated, what education is available
- Should be measured and evaluated
- Should be treated like a proper thing

Communication must be consistent.

- Should be well applied at all points of a patient’s journey

Communication helps build respect.

- Awareness of cultural diversity and different languages
- Use simple language, which isn’t just dumbed down English
- Paying extra attention
- ‘Easy English’ – incorporation of pictures

#### Shared decision-making

- Preferences, partnerships trust advocacy, shift in power relationships, recognition of wide range of decisions, high quality information, context
- Thought of in temporal terms, some open pathways
- Outcomes may vary from those that health professions think are important
- Admission of uncertainty
- Social, physical and psychosocial outcomes
- Doctors need to share decision-making in multidisciplinary teams
- Where, in clinical reviews, all options should be considered
- Think about family carers and others around patient
- Sources of evidence and data
- Give people time to make decisions
- Performance systems need to incorporated shared decision making
- Note – no acronyms

#### Shared Care Planning

- Not just hospital, but connected to community
- All health professionals know your situation, your treatment

Who are most affected;

- Carers, medical staff, health professionals need to adapt to new system
- Also change for patient, allow carers access to information
- All health professionals should be involved in plan, more holistic care
- Entire health system needs to be aware and involved

What does success look like?

- Technology infrastructure already there, people could allow access to better inform treatment options and plans, may shorten waiting lists, preventative rather than reactive care
- Health care that you need rather than starting at a point with no clear line
- Possible reduction in readmissions, people know more about looking after themselves, engagement with GPs
- All hospitals have to work together, individual systems prevent coordinated care, single source of records

#### Health and Health Systems Literacy

Being admitted to hospital is like walking into a new country

- I don’t know where to find information, where to go or who to ask

Even the term ‘health literacy’ has a judgement call

- Only some people know but some don’t
- There is a need to rephrase what health literacy is
- It is about enabling people through equity and access
- It is the continuity from start to end, and creating real understanding as a patient for what this means.

Who are most affected – it is everyone’s problem

- Staff often don’t understand well enough to explain
- Patient trying to get enough information to make a decision but struggle to get this information
- How to impart this information to consumers

For non-English speaking backgrounds or people at different points in the journey

- Health professionals should be trained to speak in plain language
- Roadmap for a journey
- Conscious and unconscious bias
- Embedding training
- Understanding information exchange
- Enable questions
- Define sets of skills
- Being able to access
- Consumers being empowered to ask questions and access information.

#### People (not) around the patient

- Who is and isn’t around
- People who are isolated
- Different cultural communities
- Being in an incorrect group
- Understand impact on individuals and families for chronic illness groups
- Responsibility to patients but also to wellbeing of families and carers

Who are most affected:

- Patients, consumers, all staff, social workers, health service staff, advocates and cares

What does success look like?

- Knowing the patient well
- Developing relationships
- Policy, flexible
- Knowing critical information
- Where would it do the most good – in leadership
- Building policies in partnerships, believing in the policies
- Data visibility, champions,
- Patients and families being empowered
- Same level of understanding

# Appendix 5: Responses to “Do you have any other thoughts, comment, reflections or ideas?” from the summit participant feedback survey

**Information sharing**

- Great day, really enjoyed it and it went very quickly. I have hope that the health system will improve and that consumers are being "heard" and their contributions valued. Patient and carer involvement are integral to create a "responsive health system".
- Very, very personal stories should maybe be put in a box if people are happy sharing difficult experiences.

**Group discussion**

- Hold more of these ideas groups.

**Consumer focus**

- Thankyou. It was great to get the opportunity to provide the consumer voice and partner with SCV to continue the patient improvement journey.
- Good balance of consumers to program staff.
- Thank you it was great to get the opportunity to provide the consumer voice and partner with SCV to continue the patient improvement journey.

**Facilitation and structure of the day**

- I was disappointed that practical implementation strategies were not discussed.
- You managed to bring together a strong and effective group. The "direct invitation" option appears to have worked well in this regard.

**Logistics of the day**

- Venue chairs were painful to sit in which became a real issue as the day went on.
- Noise control for round table discussions - difficult to moderate but very difficult to hear even the person right next to me.
- Coffee machine required a PhD

**Next Steps**

- Thankyou for the opportunity to contribute, these are very complex issues that are culturally important. The passion in the room is testament to how important they are. It was great to bring consumers and health service representatives together. I look forward to the next steps.
- I look forward to see where this goes. I will be looking to see :-)
- How will this progress and how will the participant be informed? Thank you. Great Day.
- Email. Keep the conversation going.
- I appreciate that notes will be distributed to everyone. It would be interesting to get updates on the progress of the project.
- Hopefully we will receive a summary of the day and an outline of the next steps, projects etc. Was happy to have been given the opportunity to participate in today’s session.
- Will there be a follow up session.
- Possibility of checking in again regarding planning and progress.

**Other comments**

- There are always many sides of every concern e.g. patient directed, personal care so it is not easy to land on a few effective, efficient and sustainable solutions or evolving solutions.
- This is exactly the sort of concepts and views that need attention to achieve meaningful improvement to our system and ultimately the care we deliver. Grateful to have the opportunity to contribute.
- Valuing the role that advocates can play in the experience of chronic illness patients.
- Thanks Belinda for asking me she has been so helpful, warm and made me feel really included.
- Thanks so much for this opportunity.
- A well constructed and attended day. With great contribution by all. Well done! SCV ✓✓
- I hope that the information shared today will result in making a patients experience in the health system will be improved.
- Belinda has a fantastic energy and attitude. She showed compassion and understanding when facilitating the table, which was made up of consumer reps. She skilfully extracted and analysed the information presented.
